# Anatomopathological Aspects of Acute Chagas Myocarditis by Oral Transmission

**DOI:** 10.5935/abc.20160110

**Published:** 2016-07

**Authors:** Dilma do Socorro Moraes de Souza, Marialva TF Araujo, Paulo da Silva Garcez, Julio Cesar Branco Furtado, Maria Tereza Sanches Figueiredo, Rui M.S. Povoa

**Affiliations:** 1 Faculdade de Medicina da Universidade Federal do Pará (UFPA), Belém, PA - Brazil; 2 Fundação Pública Estadual Hospital de Clínicas Gaspar Vianna, Belém, PA - Brazil; 3 Universidade Federal de São Paulo (UNIFESP), São Paulo, SP - Brazil

**Keywords:** Chagas Cardiomyopathy / pathology, Chagas Disease / transmission, Trypanosoma cruzi, Food Parasitology

## Introduction

Vector transmission of *Trypanosoma cruzi* has declined in Latin America, which has been attributed to better epidemiological control of this form of transmission, estimated at 8 to 10 million chronic cases, in addition to reducing the number of new cases.^1,2^ However, there has been an increase in the incidence of acute cases, predominantly by oral transmission due to the ingestion of food contaminated with feces of triatomids, both in isolated cases and in family micro-outbreaks.^3^

Necropsy studies that describe myocarditis in the acute phase of Chagas' disease are scarce and the existing reports in the literature are of studies carried out in the past decades and involve vector transmission in endemic areas.

Cardiac involvement in the acute phase may have varied aspects, especially in relation to myocardial lesion, from an undetectable one, to the evolution to acute heart failure with severe myocarditis and death. Cardiac involvement is present in 90% of the cases, manifesting with myocarditis and pericardial effusion.^4,5^ The analysis of a series of acute myocarditis cases showed a mortality of 5.6%.^6^

## Case Report

A 34-year-old African-descendant male patient, born and raised in the urban area of the municipality of Bragança (state of Para, Brazil), an area considered endemic for Chagas' disease was assessed.

He had had fever for 30 days, associated with chills, holocranial headache and myalgia. Three weeks before admission, he noticed dark-colored urine and dyspnea, which was progressive and quickly developed to dyspnea at minimal effort, accompanied by abdominal pain, nausea and vomiting, jaundice and facial edema. On admission, he had severe dyspnea, cold extremities, mild dehydration and mucocutaneous pallor. He was neither diabetic nor dyslipidemic. He denied hypertension and smoking. The patient regularly consumed acai juice during meals. He lived in a brick house.

On clinical examination, the heart sounds were muffled and he had tachycardia with gallop rhythm. Blood pressure was 74/40 mmHg. The lungs had decreased sounds bilaterally and the abdomen was flaccid and distended, painful to deep palpation, with no visceromegalies. Pulses were palpable, with poor peripheral perfusion and cyanosis.

Biochemical assessment on the first day of hospitalization were: TB of 3.19 mg/dL; DB of 0.18 mg/dL; IB of 2,51 mg/dL; CK of 537 U/L; CKmb of 139 U/L; Hemoglobin (Hb) of 12 g; Hematocrit (Htc) of 32%; leukocytes of 15,600; lymphocytes of 30,000; platelets of 161,000/mm³; TGO of 860 U/l; TGP of 421 U/L; PT of 25; APTT of 53.

ECG showed junctional tachycardia, low-voltage complexes in classical leads and diffuse ventricular repolarization alterations.

Chest x-ray showed significant cardiomegaly with left pleural effusion, and the Doppler echocardiogram showed LVDD of 58 mm, LVSD of 39 mm, IVS of 10 mm, LVPW of 10 mm. The EF was estimated at 23% LA of 41 mm; with diffuse hypocontractility. The valves were normal and there was mild mitral, aortic and tricuspid regurgitation. Severe pericardial effusion was also observed.

The thick smear examination was positive for*T. Cruzi*.

On the second day of hospitalization, he developed fever (38 C), with worsening of dyspnea and required pericardial effusion drainage. He underwent endotracheal intubation and developed septic and cardiogenic shock and ventricular arrhythmias. He was treated with benznidazole, Angiotensin Converting Enzyme (ACE) inhibitors, beta-blockers, vasoactive amines, ceftriaxone and amiodarone. He went into cardiac arrest, which was refractory to resuscitation maneuvers and died.

The cause of death was septic shock and cardiogenic shock. The anatomopathological diagnosis of the necropsy study was described as heart failure due to acute Chagas myocarditis.

### Anatomopathological Heart Study

**Figure 1:**
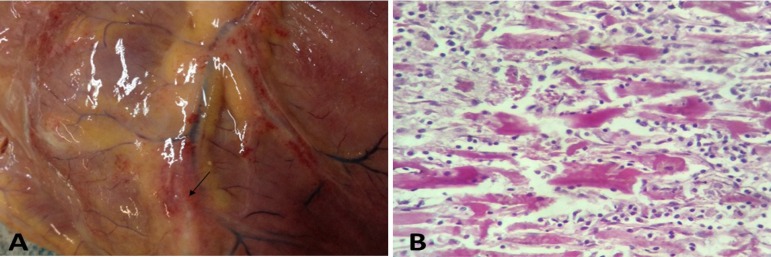
Macroscopic aspects of the heart, showing a flaccid, globular heart with smooth and shiny aspect (A). Extensive lytic hyaline destruction, mononuclear cell infiltrate, with areas of cardiac fiber dissociation and necrosis (B).

At the macroscopic analysis, the heart was globular, pale-gray, flaccid and congested. The presence of pericardial fluid was above normal. The assessment of the cardiac chambers showed significant chamber dilation (with predominantly dilated left chambers, mainly the left ventricle). The myocardial study showed a pinkish-gray and shiny aspect; the parietal endocardium was smooth and shiny. The heart valves were normal. The epicardium was smooth and shiny. Following the trajectory of the coronary branches, a number of small nodules was observed, described in previous studies as "rosary beads" (moliniform epicarditis),^7^ as shown in Figure 1A.

The microscopic study showed severe acute inflammation of the epicardium and myocardium, preserving the endocardium and normal coronary arteries.

The myocardium showed intense and diffuse inflammatory activity, extensive cardiac fiber dissociation and massive destruction of the entire cardiac tissue (Figure 1B).

**Figure 2:**
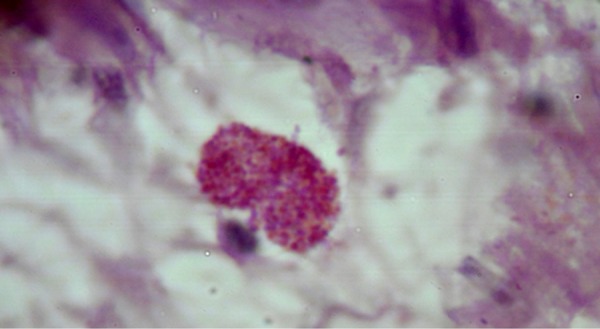
Amastigote in the cardiac myocyte with clear halo and vacuolated mitochondria.

The histological sections, which were stained with hematoxylin-eosin and Giemsa, disclosed the parasite-containing amastigotes (Figure 2).

### Comments

The case described here reports on a young patient who acquired Chagas disease by oral transmission in a family micro-outbreak attributed to acai juice consumption. The case was considered as oral transmission due to the epidemiological evidence of the acai consumption and the fact that two relatives had concomitant symptoms without entry signs, considered classical of vector transmission.^8^ In the Amazon region, the incidence of acute Chagas disease has increased since 1996, especially in the state of Para. Over a thousand acute cases were documented, caused by family micro-outbreaks, between the years 2000-2010, and most (71 %) of these cases were attributed to probable contamination of food with triatomid feces.^9^ Acute cases of the disease occur throughout the year, but a higher incidence is observed in the second semester, especially in the months of September and October, which coincides with the time of acai harvest in the region.^5^

The possible mechanisms of transmission of *T. cruzi* by oral route are attributed to the ingestion of food contaminated with feces of triatomids or urine of marsupials, as well as eating infected undercooked meat of wild animals. These conditions are facilitated by the fact that the parasite survives at temperatures similar to that of the vector, especially in tropical regions, with high humidity and environment temperature, favoring this form of transmission.^10^ Acai is the most often consumed fruit, and its palm tree is found both in the vicinity of residences, as well as in forested areas. The combination of poor hygiene facilitates this route of infection, despite the presence of the classic vector responsible for the usual form of transmission.^11^

Generally, oral *T. cruzi* infection has an incubation period ranging from 3 to 21 days and, when symptoms are apparent, it starts with fever, which is usually prolonged, lasting on average 19 days. This form of transmission is considered the most severe one, a fact observed in our patient.^8^ Animal models of acute infection by *T. cruzi*, correlated the aggressiveness of clinical manifestations, mainly the cardiac abnormalities, with a high parasite load and to high *inoculum*.^12^ Consistent with these studies, we considered the possibility that our patient ingested a large *inoculum* and, thus, had a more severe clinical evolution when compared to his relatives.

Acute myocarditis by *T. cruzi* can be fatal in 3-5% of cases and is the leading cause of death after meningoencephalitis.^13^ The junctional rhythm, which develops into ventricular fibrillation, significant ventricular dysfunction and pericardial effusion, manifested by the patient, is similar to the finding described in myocarditis by other etiologies. Marques et al.^14^ reported the presence of arrhythmias in 26.5% of cases of oral infection by *T. cruzi*. Ventricular repolarization alterations and low-voltage complexes are common in acute Chagas myocarditis and are present in the initial electrocardiogram.^15^

We report the finding of inflammatory nodules in the coronary trajectory, described in rare literature records as moliniform epicarditis or "rosary beads", which usually appear after 30 days of infection, coinciding with our patient's disease evolution stage and in accordance with reports in the literature regarding the stage of the disease.^7^

An interesting aspect of the histological study was the massive destruction of cardiac cells, a fact that was not described in histological studies of vector transmission, which leads to the assumption about the severity of the oral transmission route.^15^ We stress the finding of ruptured cysts of amastigotes. Torres et al.^16^ had already described finding these cysts, containing intracellular forms of amastigotes of Leishmania, in 1948, present in the cardiac tissue during the acute phase of *T. cruzi* infection.

In this case, the aggressiveness of the acute cardiac lesion involving the oral route of transmission is quite evident. We conclude that myocarditis can be extremely severe and fatal, and the implementation of public policies directed at food hygiene strategies must be implemented immediately in order to minimize the globalization of disease by this route of transmission.

The association between acai consumption and Chagas disease have been increasing annually in Para, especially in cities where there is a significant number of acai sales points, which results in increased supply of the drink throughout the year and especially during the time of the harvest, making these points a "common source", that is, the single interrelated factor among the cases.
