# Erratum

**DOI:** 10.5935/abc.20170038

**Published:** 2017-03

July issue of 2016, vol. 107 (1), pages. 77-80

In the Case Report “Anatomopathological Aspects of Acute Chagas Myocarditis by Oral Transmission”, pages 77-80, by authors Dilma do Socorro Moraes de Souza, Marialva TF Araujo, Paulo da Silva Garcez, Julio Cesar Branco Furtado, Maria Tereza Sanches Figueiredo, Rui M.S. Povoa, please be aware that the correct spelling for Paulo da Silva Garcez is Paulo Roberto Silva Garcez dos Santos.

